# Subnanometer-Wide Indium Selenide Nanoribbons

**DOI:** 10.1021/acsnano.3c00670

**Published:** 2023-03-14

**Authors:** William J. Cull, Stephen T. Skowron, Ruth Hayter, Craig T. Stoppiello, Graham A. Rance, Johannes Biskupek, Zakhar R. Kudrynskyi, Zakhar D. Kovalyuk, Christopher S. Allen, Thomas J. A. Slater, Ute Kaiser, Amalia Patanè, Andrei N. Khlobystov

**Affiliations:** † School of Chemistry, University of Nottingham, Nottingham, NG7 2RD, United Kingdom; ‡ Nanoscale and Microscale Research Centre, University of Nottingham, Nottingham NG7 2QL, United Kingdom; § Central Facility of Electron Microscopy, Electron Microscopy Group of Materials Science, University of Ulm, 89081 Ulm, Germany; ∥ School of Physics, University of Nottingham, Nottingham NG7 2RD, United Kingdom; ⊥ Faculty of Engineering, University of Nottingham, Nottingham NG7 2RD, United Kingdom; # Institute for Problems of Materials Science, National Academy of Sciences of Ukraine, Chernivtsi Branch, 58001 Chernivtsi, Ukraine; ∇ Electron Physical Sciences Imaging Centre, Diamond Light Source ltd, Didcot OX11 0DE, United Kingdom

**Keywords:** III−VI semiconductor, indium selenide, phase change material, nanoribbons, nanowires, carbon nanotubes

## Abstract

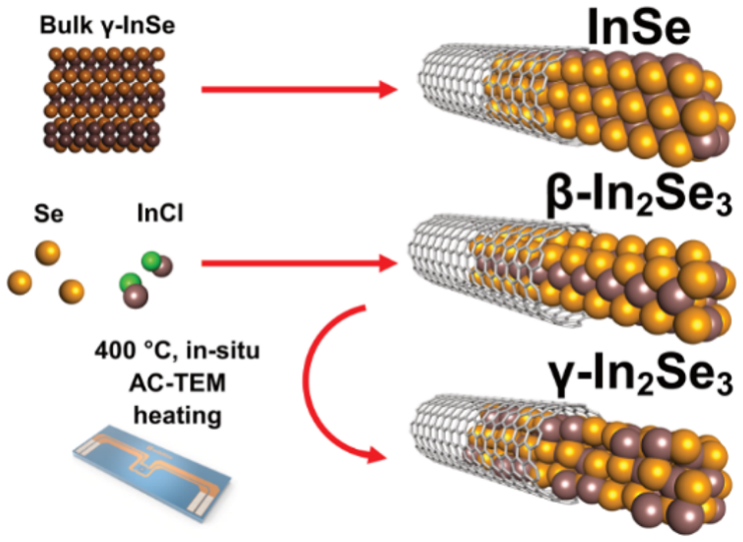

Indium selenides (In_*x*_Se_*y*_) have been shown to retain several desirable properties, such as ferroelectricity, tunable photoluminescence through temperature-controlled phase changes, and high electron mobility when confined to two dimensions (2D). In this work we synthesize single-layer, ultrathin, subnanometer-wide In_*x*_Se_*y*_ by templated growth inside single-walled carbon nanotubes (SWCNTs). Despite the complex polymorphism of In_*x*_Se_*y*_ we show that the phase of the encapsulated material can be identified through comparison of experimental aberration-corrected transmission electron microscopy (AC-TEM) images and AC-TEM simulations of known structures of In_*x*_Se_*y*_. We show that, by altering synthesis conditions, one of two different stoichiometries of sub-nm In_*x*_Se_*y*_, namely InSe or β-In_2_Se_3_, can be prepared. Additionally, *in situ* AC-TEM heating experiments reveal that encapsulated β-In_2_Se_3_ undergoes a phase change to γ-In_2_Se_3_ above 400 °C. Further analysis of the encapsulated species is performed using X-ray photoelectron spectroscopy (XPS), thermogravimetric analysis (TGA), energy dispersive X-ray analysis (EDX), and Raman spectroscopy, corroborating the identities of the encapsulated species. These materials could provide a platform for ultrathin, subnanometer-wide phase-change nanoribbons with applications as nanoelectronic components.

Indium selenides (In_*x*_Se_*y*_) belong to a family of group III–VI semiconductors attracting increasing research interest due to their potential applications as ultrathin and flexible components of photovoltaic and optoelectronic devices.^1,2^ These materials are known to exist in a great variety of stoichiometric ratios, and among these, different phases, all with differing chemical and physical properties. Multiple studies have been conducted into the interconversion of the stoichiometries, phases, and stacking of In_*x*_Se_*y*_ compounds, often with contradictory results, as summarized by Han *et al*.^3^ If their full potential is to be realized, an understanding of the complex polymorphism of In_*x*_Se_*y*_ must become established. Beyond the known members of the In_*x*_Se_*y*_ family, swarm-intelligence-based computational studies predict the existence of experimentally undiscovered stable polymorphs of InSe, indicating the importance of further research into controlling the structure of In_*x*_Se_*y*_ compounds.^4^

Indium sesquiselenide (In_2_Se_3_) is known to exist in five different phases, with the most common being the α, β, and γ phases.^3,5−8^ In α-In_2_Se_3_ half of the In atoms are octahedrally coordinated and half are tetrahedrally coordinated, whereas in β-In_2_Se_3_ all In atoms are octahedrally coordinated. Both α- and β-In_2_Se_3_ phases are layered structures, with van der Waals bonds separating layers in the direction of the *c* axis and bulk phases of both known to undergo irreversible phase transitions to γ-In_2_Se_3_ at high temperatures. The exact temperature required for this transformation is contentious but has been shown to occur between 350 and 650 °C.^9,10^ Unlike its α and β counterparts, γ-In_2_Se_3_ is not a layered structure, instead forming a defected wurtzite crystal structure. Indium monoselenide (InSe) can exist in three different phases, β, ε, or γ, determined by the stacking of van der Waals layers in the structure.^11^ The structures of bulk β-In_2_Se_3_, γ-In_2_Se_3_, and γ-InSe are shown in Figure 1.^7,8,12,13^

**Figure 1:**
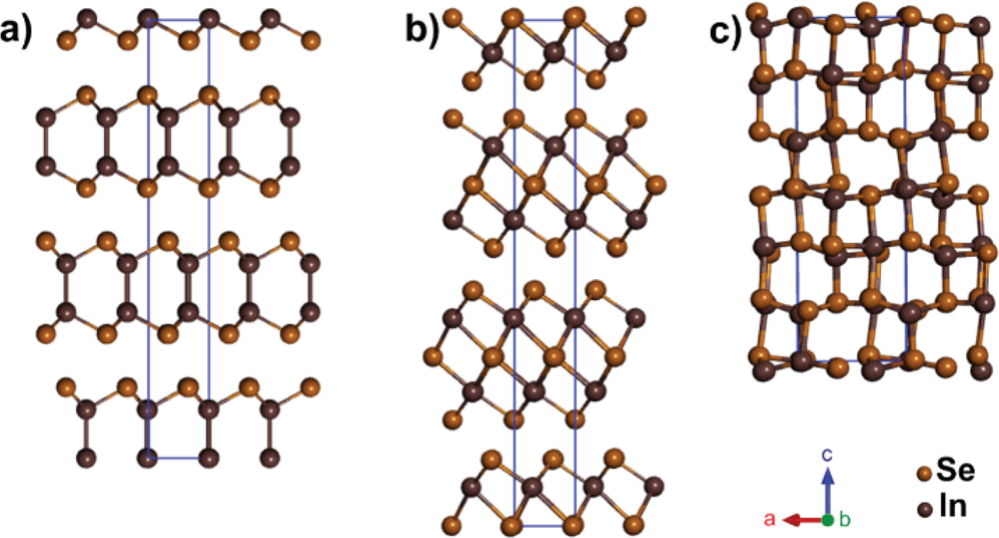
Structures showing the (120) plane of bulk (a) γ-InSe, (b) β-In_2_Se_3_, and (c) γ-In_2_Se_3_ with their unit cells shown in blue.

Confining In_*x*_Se_*y*_ to two dimensions (2D), i.e. a single van der Waals layer, has been shown to alter the physical properties of the material significantly. Mudd *et al*. showed that the band gap of layered γ-InSe can be drastically modified by reducing its thickness, with further studies reporting direct-to-indirect band gap crossover.^14,15^ The observation of room- and liquid helium-temperature quantum Hall effects has also been reported in atomically thin layers of γ-InSe, indicating their potential for use in high-quality 2D semiconducting components in the future.^16,17^ Confinement of In_2_Se_3_ phases to 2D has shown that certain unique properties of the bulk material can be maintained, including room-temperature ferroelectricity and high electron mobility.^10,18^ Ferroelectricity in few- and single-layer α-In_2_Se_3_ was theoretically predicted by Ding *et al*., with subsequent experimental studies confirming the same property in few-layer α-In_2_Se_3_ and β-In_2_Se_3_.^19−22^ Despite the progress made in this field, the production of nanosized In_*x*_Se_*y*_ is typically not scalable, often relying on liquid-phase or mechanical exfoliation to construct 2D sheets. In_2_Se_3_ nanowires have been successfully synthesized, revealing that the intricate phase-change behavior of In_*x*_Se_*y*_ compounds is maintained when templated in this manner, along with a high photosensitivity and rapid photoresponse when utilized as a visible light photodetector.^23−26^ However, the nanowire diameters are much greater than that of a single van der Waals layer (measuring between 40 and 200 nm), meaning they lack some of the exciting properties of ultrathin, single-layer In_*x*_Se_*y*_. Indeed, a comprehensive literature review on low-dimensional In_2_Se_3_ conducted by Li *et al*. and published in 2021 shows that the smallest diameter In_2_Se_3_ nanowires, synthesized by Sun *et al*. in 2006, measure 40–80 nm in diameter.^23,27^ Additionally, Ho *et al*. showed that low-dimensional In_*x*_Se_*y*_ field effect transistors (FETs) deteriorate *via* oxidation under ambient conditions unless protected by a passivating hexagonal boron nitride layer, an important consideration if the widespread use of In_*x*_Se_*y*_ based nano electronic components is to be realized.^28^

Single-walled carbon nanotubes (SWCNTs) can be conceptualized as a rolled-up sheet of graphene, forming a tube-like structure with a hollow internal cavity.^29^ The highly anisotropic geometry of SWCNTs offers a unique environment for the templated growth of ultrathin nanomaterials,^30,31^ while also allowing for the encapsulated materials to be studied easily by transmission electron microscopy (TEM).^32^ Recently synthesized materials include ReS_2_,^33^ SnSe,^34^ and HgTe,^35^ each forming ultrathin nanoribbons inside the internal cavity of the SWCNTs. Nanowires and nanoribbons synthesized by encapsulation in SWCNTs have great potential as components in nanoelectronic devices due to their high aspect ratio and small diameter.^36^ SWCNTs also offer increased thermal and chemical stability to the encapsulated materials, having been shown to increase the melting point and/or prevent decomposition under ambient conditions when compared to their unencapsulated counterparts.^37,38^ Aside from providing a stable nanoscale container, the extreme confinement enforced when a molecule or molecular material is encapsulated within SWCNTs has been shown to yield unique species with geometries and bonding not seen in their bulk counterparts.^39,40^

In this study we use various synthetic methods to create different phases of ultrathin SWCNT-encapsulated In_*x*_Se_*y*_ (In_*x*_Se_*y*_@SWCNT), simultaneously producing sub-nm diameter In_*x*_Se_*y*_ nanoribbons and providing a scalable method to synthesize single-layer In_*x*_Se_*y*_. While the confinement of In_*x*_Se_*y*_ to 2D has yielded the discovery of many exciting properties, it is currently unknown which of these are retained when dimensionality is reduced even further. Aberration-corrected transmission electron microscopy (AC-TEM) analysis is used to show that the phase of the encapsulated material can be controlled by altering the experimental conditions. This, coupled with energy dispersive X-ray (EDX) analysis, X-ray photoelectron spectroscopy (XPS), AC-TEM simulations,^41^ thermogravimetric analysis (TGA), Raman spectroscopy, powder X-ray diffraction (PXRD), and differential scanning calorimetry (DSC), allows for rigorous characterization of the structure, bonding, phase, and stoichiometry of encapsulated species. Finally, we employ an *in situ* heating experiment within the TEM to show that the encapsulated material experiences a temperature-induced phase change behavior similar to that of bulk and 2D In_*x*_Se_*y*_, albeit with increased reversibility.

## Results and Discussion

### Indium Selenide Nanoribbons Formed from Melt Growth

We explored two methods for the synthesis of In_*x*_Se_*y*_ nanoribbons, both employing the use of SWCNTs with 1.5 nm diameters as a template. The first method is based on melting bulk γ-InSe in the presence of open SWCNTs. Melt growth has previously been used to successfully encapsulate a variety of compounds inside CNTs, including metal chlorides, metal oxides, and elemental species, allowing for the synthesis of numerous ultrathin nanowires and nanoribbons.^42,43^ For example, PbO, which like γ-InSe is also a layered 2D inorganic material, has previously been encapsulated in SWCNTs of a similar diameter by a melt growth method, yielding well-ordered nanoribbons of single-layer β-PbO.^44^ γ-InSe, produced using the Bridgman method from a polycrystalline melt of In and Se as described by Bandurin et al.,^17^ was found to melt at 650 °C. Opened SWCNTs were sealed in an evacuated quartz ampule with γ-InSe and heated to 800 °C. An extended reaction time of 70 h and a temperature above the melting point of γ-InSe was used so as to compensate for the high viscosity of molten γ-InSe, which was likely to reduce the kinetics of atomic diffusion into nanotube cavities, as per the Young–Laplace equation.^45,46^

**Figure 2:**
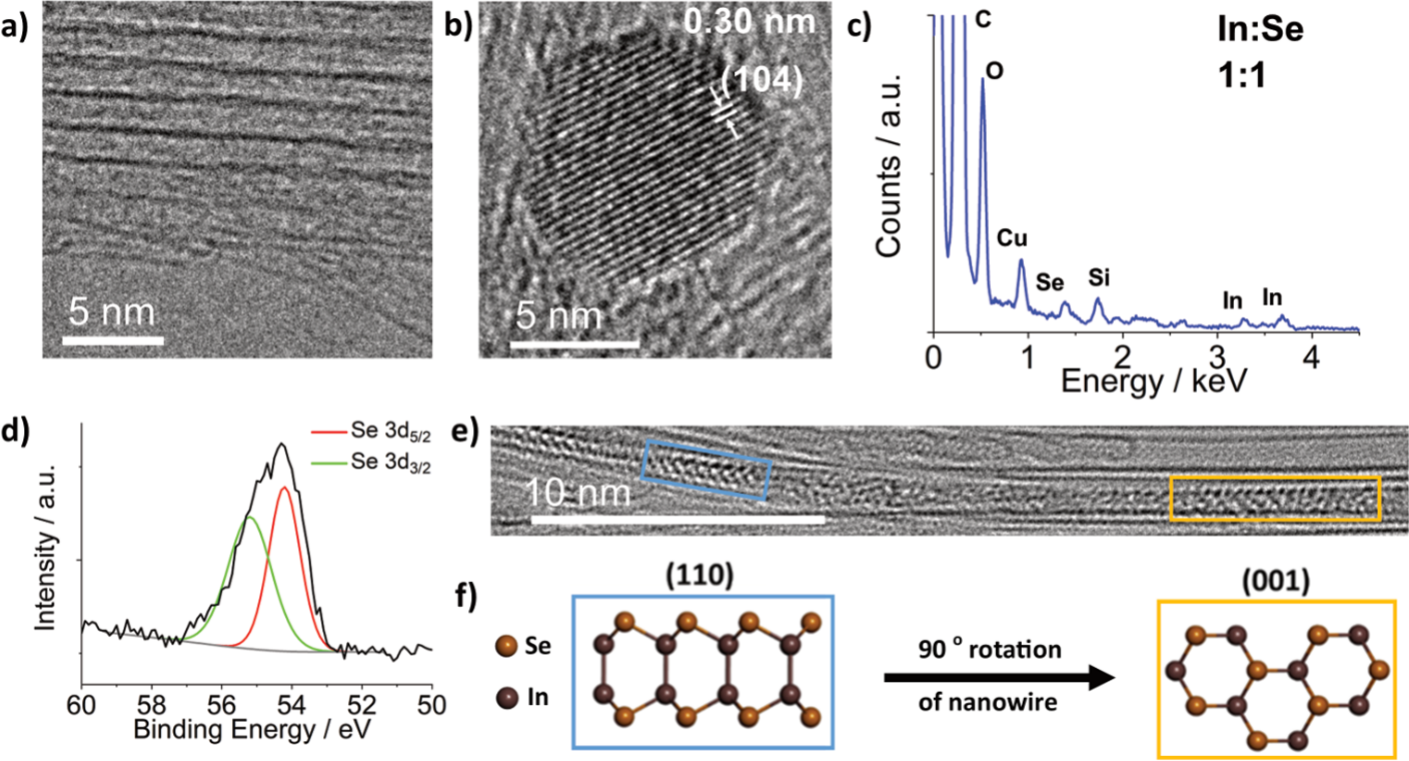
Experimental results from the melt growth of γ-InSe inside SWCNTs: (a) 200 kV TEM of InSe@SWCNT; (b) 200 kV TEM of externally bound γ-InSe nanoparticle; (c) EDX spectrum of the area shown in (a); (d) XPS spectrum of InSe@SWCNT showing the Se 3d environment (e) 80 kV AC-TEM image of a single InSe nanoribbon inside a SWCNT with two distinct orientations highlighted in blue and yellow boxes; (f) molecular models created from single-layer InSe showing the proposed appearance of the two distinct orientations seen in (e), the (110) plane shown in the blue box and the (001) plane shown in the yellow box.

For the material prepared by melt growth, transmission electron microscopy (TEM) analysis revealed externally bound γ-InSe nanoparticles of up to 20 nm in diameter (Figure 2b) identified by their characteristic *d* spacing (0.30 nm, (104)).^12^ Energy dispersive X-ray (EDX) analysis of SWCNT bundles with no external γ-InSe nanoparticles (Figure 2c) indicated In and Se in a 1:1 ratio. This was consistent with X-ray photoelectron spectroscopy (XPS) analysis, showing 3d_3/2_ and 3d_5/2_ photoelectron lines for both In and Se (Figures S1 and 2d, respectively). In comparison to photoelectron lines for bulk γ-InSe (Figures S2 and S3), In and Se 3d_5/2_ peaks were found to red-shift by 0.4 and 0.3 eV, respectively. This shift is attributed to the SWCNTs being able to donate electron density to InSe. Despite this shift, the binding energies observed are within the expected range of In^2+^ and Se^2–^, further confirming that any encapsulated InSe retained its stoichiometry following the melt growth.^47^

Because of their extremely narrow width and lack of 3D periodicity, the traditional bulk characterization methods of spectroscopy and diffractometry proved inconclusive for the encapsulated nanoribbons. Therefore, we applied local probe microscopy methods, including conventional HRTEM and AC-HRTEM, to determine the structure and composition of the In_*x*_Se_*y*_ nanoribbons. The atomically thin sidewalls of the SWCNTs are easily penetrated by the electron beam of a transmission electron microscope (TEM), allowing for the acquisition of atomically resolved direct-space images. Conventional HRTEM analysis of the filled SWCNTs revealed encapsulated nanoribbons with a high degree of translational mobility within the SWCNT internal cavity, due to the influence of the electron beam, as shown in Figure 2a, making it difficult to determine the exact atomic positions, and therefore the chemical structure, of the encapsulated nanoribbons. Such fast translational motion has not been reported for metal sulfide nanoribbons in carbon nanotubes.^48^ It is likely to be related to repulsive interactions between the terminal Se atoms on the edges of the InSe nanowire and the carbon atoms of the SWCNT internal cavity, as predicted by Fujimori *et al*. for Se chains on graphitic carbon.^49^ However, it was found that the encapsulated nanoribbons could be atomically resolved by either increasing the capture rate of TEM imaging or finding areas where the nanoribbons were pinned in place by small defects in the SWCNT sidewalls. AC-HRTEM of these areas afforded atomically resolved images of the encapsulated nanoribbons, revealing the structure of the encapsulated species. It is important to note that the phase of InSe (e.g., β, ε, or γ) only determines differences in stacking between multiple van der Waals layers of the crystal. As a result, classification of the exact phase of any encapsulated InSe is redundant if the encapsulated nanoribbon is made up of a single monolayer, as it is here.

**Figure 3:**
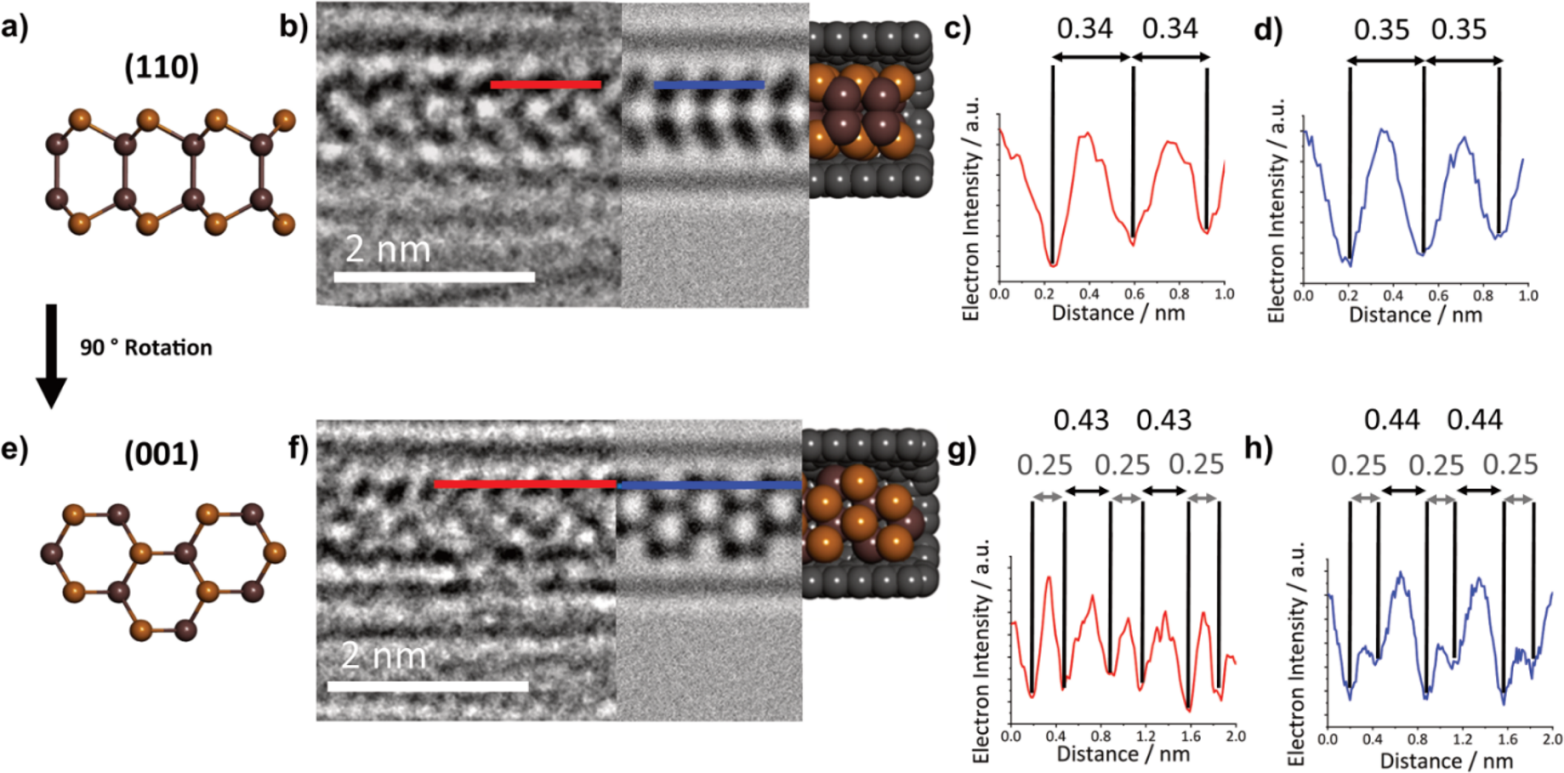
(a) Structural model of monolayer InSe in the (110) orientation. (b) Three-part composite image of an InSe nanoribbon inside a SWCNT, consisting of an AC-TEM image (left), a simulated TEM image (center), and molecular model (right). (c, d) Electron density profile maps in red, generated from the red line superimposed over the experimental AC-TEM image in (b), and in blue, generated from the blue line superimposed over the simulated TEM image in (b), with calculated interatomic distances highlighted in nm. (e–h) The same as (a–d), respectively, but for the (001) orientation of the same nanoribbon.

The AC-TEM image shown in Figure 2e displays a nanoribbon with an appearance in projection similar to that of a monolayer of γ-InSe viewed down the (110) plane, as highlighted by the blue box. The similarities in appearance were further probed via comparative contrast analysis of experimental and simulated TEM images of the (110) plane of InSe@SWCNT, revealing very similar spacing (0.34 and 0.35 nm, respectively) between columns of atoms (Figure 3b–d). In addition, the width of the (110) plane nanoribbon was measured experimentally as 0.67 nm, agreeing well with the simulated width of 0.68 nm. The specific nanoribbon shown in Figure 2e undergoes a 90° twist around the SWCNT axis, showing a projection like that of the (001) plane of a monolayer of γ-InSe, as highlighted by the yellow box. TEM simulations were used to confirm this similarity, with a matching diameter (0.62 nm experimental and 0.62 nm simulated) and alternating atomic spacing shown in both the simulated and experimental images, as shown in Figure 3f–h. The close match between the simulated and experimental TEM images for the two distinct orientations of the same nanoribbon is compelling evidence for the successful encapsulation of monolayer InSe inside SWCNT.

### Indium Selenide Nanoribbons Formed from Stepwise Synthesis

**Scheme 1:**
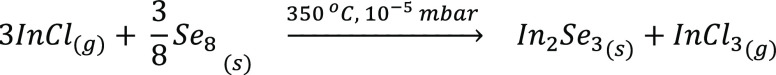
Reaction to Form Indium Sesquiselenide

The second method is a stepwise nanoribbon synthesis, performed by sequentially introducing selenium and indium precursors into nanotubes, similar to the previously reported synthesis of SWCNT-encapsulated metal sulfides.^48^ Recent work by Huang *et al*. showed that InSe nanoflakes could be synthesized from Se and InI vapor, utilizing an Ar and H_2_ carrier gas.^50^ The H_2_ included in the carrier gas mix was shown to promote the production of InSe over In_2_Se_3_, along with inducing the growth of multiple layers of InSe. In order to synthesize In_*x*_Se_*y*_@SWCNT, metallic Se and InCl were chosen as Se and In precursors, respectively. InCl was selected instead of InI as it was found to possess a higher solubility in conventional solvents, such as water and THF, allowing for externally adsorbed precursors to be readily removed in subsequent washing steps. InCl and Se were found to sublime at 350 and 550 °C, respectively, when sealed under a 10^–5^ mbar vacuum. Additionally, a hydrogen carrier gas was not employed, due to there being no need to grow multiple layers of In_*x*_Se_*y*_, while simultaneously inducing the formation of In_2_Se_3_ over InSe. First, selenium was introduced into SWCNTs, followed by the selective removal of excess selenium from the external surface of the nanotube by washing with CS_2_. Next, InCl was sublimed into nanotubes, inducing the following reaction shown in Scheme 1 with SWCNT encapsulated Se.

**Figure 4:**
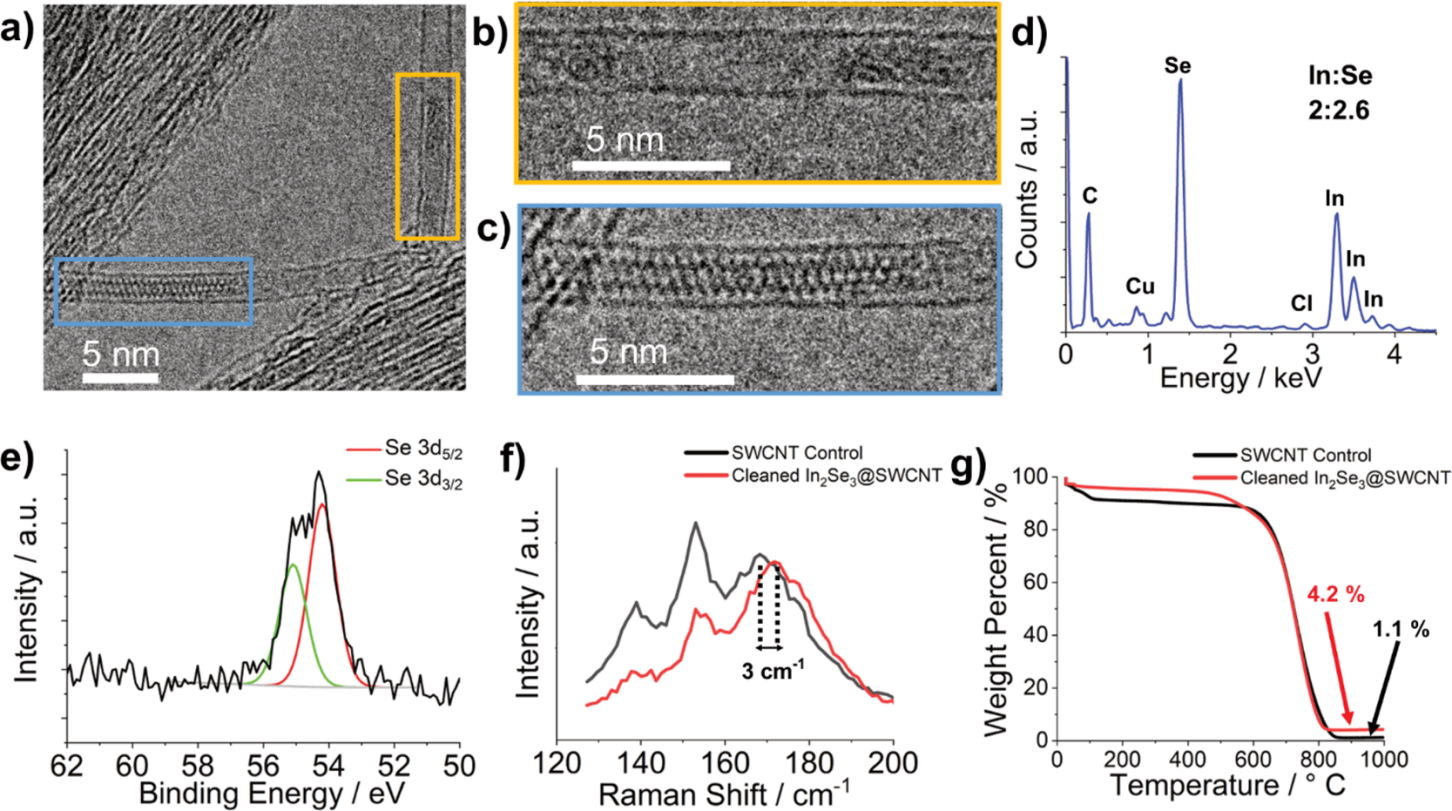
(a) 200 kV TEM image of In_2_Se_3_@SWCNTs. (b, c) Digitally magnified TEM images of the yellow and blue areas, respectively, highlighted in (a). (d) EDX spectrum of a bundle of In_2_Se_3_@SWCNTs. (e) XPS spectrum of In_2_Se_3_@SWCNTs showing the Se 3d environment. (f) Raman analysis of In_2_Se_3_@SWCNTs (red) and control SWCNTs (black), showing the clear blue shift in the position of the RBM of the smallest diameter metallic SWCNTs, resonant with the 660 nm excitation laser following encapsulation. (g) Thermogram of In_2_Se_3_@SWCNTs (red) and control SWCNTs (black) in air, showing the difference in residual weight following heating to 1000 °C.

Further cleaning steps, including sublimation cleaning and acid washing, were then employed to remove externally bound precursors and byproducts (details are given in the Experimental Section), followed by a final high-temperature annealing step. This stepwise method afforded In_*x*_Se_*y*_@SWCNT with a clean external nanotube surface, as shown by TEM and PXRD analysis (Figure 4a and Figures S4 and S5). A control sample of empty SWCNTs was prepared under the same reaction conditions but without Se and InCl, to account for any changes to the structure of the SWCNTs induced during the multistep reaction.

Much like the encapsulated nanoribbons grown from melt growth, a conventional TEM analysis of In_*x*_Se_*y*_ nanoribbons prepared by stepwise synthesis revealed the highly translational mobility of the encapsulated nanoribbons. Most of the encapsulated species were found to be too mobile to discern the exact atomic positions (Figure 4b), with only a small number of immobilized nanoribbons enabling structural information about the encapsulated species to be revealed (Figure 4c). EDX analysis of filled SWCNTs revealed peaks corresponding to In, Se, and Cl in atomic abundance ratios of 2:2.6:0.035, respectively. This atomic ratio implies that the encapsulated In_*x*_Se_*y*_ is likely to be predominantly of the In_2_Se_3_ stoichiometry, with a trace amount of Cl remaining even after cleaning. Similarly, an XPS analysis confirmed both Se and In photoelectron lines (Figure 4e and Figure S7, respectively), in a single environment, indicating a single phase of In_2_Se_3_. Additionally, when compared to the position of the Se 3d_5/2_ photoelectron line of Se@SWCNT at 55.3 eV (Figure S6), the Se 3d_5/2_ photoelectron line of In_2_Se_3_@SWCNT was found at 54.3 eV. This decrease in photoelectron binding energy is indicative of the reduction of Se following reaction with InCl. In contrast, In 3d_5/2_ photoelectron lines do not show a shift in energy after the reaction to form In_2_Se_3_, due to In^+^ in InCl and In^3+^ in In_2_Se_3_ having indistinguishable photoelectron binding energies.^47^

Unlike the melt growth method, the external surface of these SWCNTs could be cleaned thoroughly. This allowed for further analysis of the sample by bulk techniques, including TGA and Raman spectroscopy, affording further information about the encapsulated In_2_Se_3_. Raman spectroscopy of the cleaned nanoribbons prepared by stepwise synthesis showed a blue shift in the nanotube’s radial breathing mode (RBM) when compared to control SWCNTs (Figure 4f), implying strong van der Waals forces between the SWCNT and the confined In_2_Se_3_ nanoribbon, resulting in constriction of the nanotube diameter to maximize these attractive interations.^51^ Thermogravimetric analysis (TGA) of the cleaned In_2_Se_3_@SWCNT and control SWCNTs in air both showed two thermal events of interest: first, evaporation of water from the internal cavity of the SWCNTs between 40 and 100 °C, and second, the combustion of the SWCNTs between 600 and 800 °C. The SWCNTs filled with indium selenide nanoribbons were found to contain less encapsulated water and a higher residual weight after nanotube combustion than empty SWCNTs, both attributed to the presence of encapsulated In_2_Se_3_. Additionally, the onset of nanotube combustion for the filled SWCNTs is around 50 °C lower than for empty SWCNTs, which may be explained by the encapsulated material catalyzing the combustion of the SWCNTs, as seen previously in the combustion of graphitized nanofiber encapsulated MoO_2_.^52^ The SWCNT likely acts as a protective sheath to the encapsulated In_2_Se_3_, protecting the anisotropic nanoribbon from oxidation, as seen by Bendall *et al*. with SWCNT-encapsulated metal halides being much more thermally stable than their unencapsulated counterparts.^53^ Powder X-ray diffraction (PXRD) analysis of cleaned In_2_Se_3_@SWCNTs (Figure S5) showed no peaks corresponding to externally bound precursors, only those corresponding to SWCNTs.^54^ PXRD analysis of the material afforded after TGA indicated the presence of NiO (residual catalyst from nanotube growth) and In_2_O_3_, a combustion product of In_2_Se_3_ (eq S1). It can be assumed that 1.1% of the residual mass of TGA combusted residual weight relates to NiO. Assuming complete combustion, this corresponds to a 5.0 wt % loading of In_2_Se_3_ inside the SWCNTs.^55^ This percentage loading is similar to values seen for other CNT-encapsulated species.^56,57^

**Figure 5:**
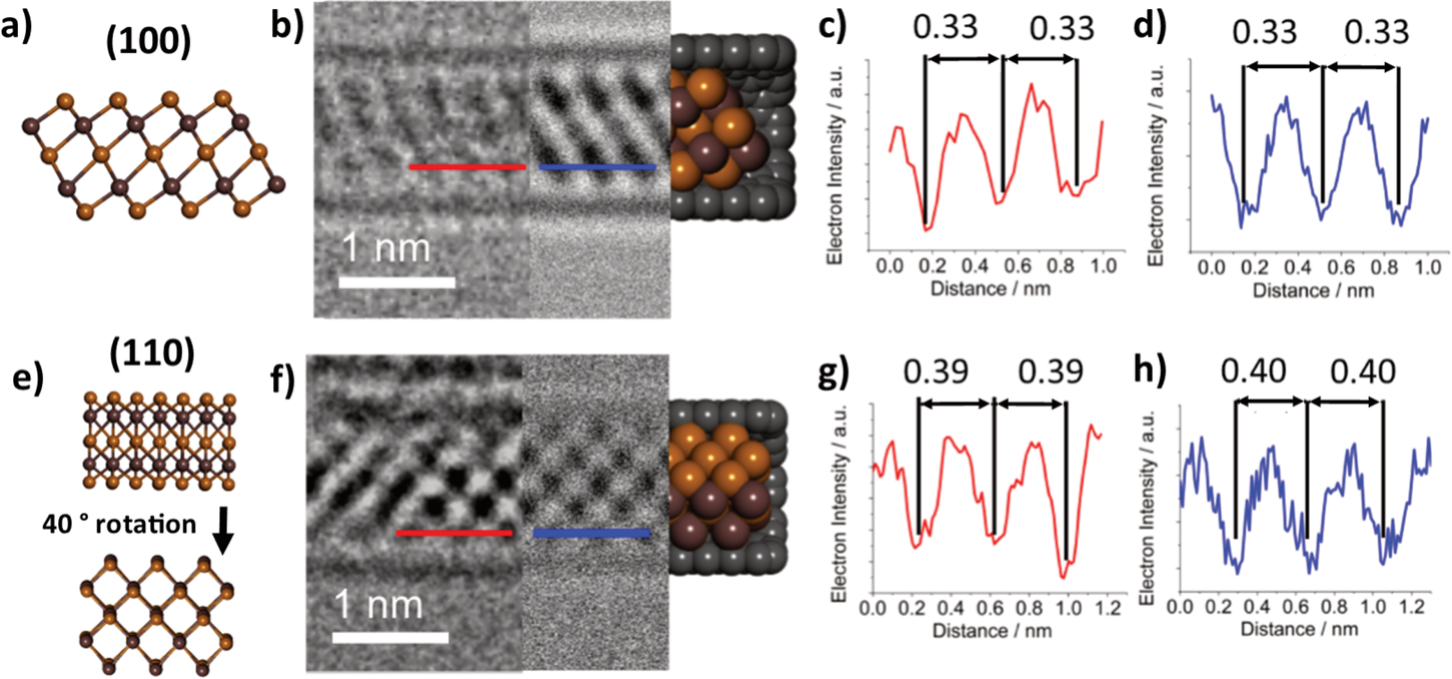
(a) Structural model of monolayer β-In_2_Se_3_ in the (100) orientation. (b) Three-part composite image of an InSe nanoribbon inside a SWCNT, consisting of an AC-TEM image (left), a simulated TEM image (center), and molecular model (right). (c, d) Electron density profile maps in red, generated from the red line superimposed over the experimental AC-TEM image in (b), and in blue, generated from the blue line superimposed over the simulated TEM image in (b), with calculated interatomic distances highlighted in nm. (e–h) The same as (a–d), respectively, but for a different nanoribbon in the (110) orientation.

To determine the exact phase of the encapsulated In_2_Se_3_, an AC-HRTEM analysis was performed, allowing for atomically resolved images of single nanoribbons to be obtained. Figure 5b shows a nanoribbon with appearance, atomic spacing, and nanoribbon diameter very similar to that of a simulated monolayer of β-In_2_Se_3_ viewed along the (110) plane and propagating in the (100) plane. Other orientations of encapsulated β-In_2_Se_3_ were also seen, including a nanoribbon with an appearance like the (100) plane monolayer β-In_2_Se_3_, rotated 40° around the axis of the SWCNT (Figure 5f), propagating in the (120) plane. From this, it is clear that encapsulated monolayer β-In_2_Se_3_ has the freedom to propagate in multiple directions. It is also important to note that these β-In_2_Se_3_ nanoribbons still possess the noncentrosymmetric structural motif required for room-temperature in-plane ferroelectricity in few-layer β-In_2_Se_3_, as stated by Zheng *et al*.^21^

### Thermally Induced Phase Transition from β-In_2_Se_3_ Nanoribbon to γ-In_2_Se_3_ Nanowire

Indium selenide is a highly diverse material, existing in many different stoichiometries and phases. Nanoribbons of β-In_2_Se_3_ encased in atomically thin, thermally conducting SWCNTs (β-In_2_Se_3_@SWCNTs) offer an opportunity to study phase transitions at the atomic level. To assess the ability of nanoconfined β-In_2_Se_3_ to undergo thermally induced phase changes, β-In_2_Se_3_@SWCNT was deposited onto a DENS Solutions heating chip, allowing for *in situ* AC-TEM imaging to be performed while the sample was heated. The sample was heated in 100 °C intervals to 500 °C from room temperature. After 60 s of heating at a specified temperature the DENS Solutions heating chip was returned to 23 °C to allow for AC-HRTEM imaging to take place. This was done to reduce the thermally induced movement of nanoribbons, offering preferential imaging conditions. The electron beam of the TEM was blanked while heating took place so as to avoid the occurrence of any beam-induced phase transitions.

**Figure 6:**
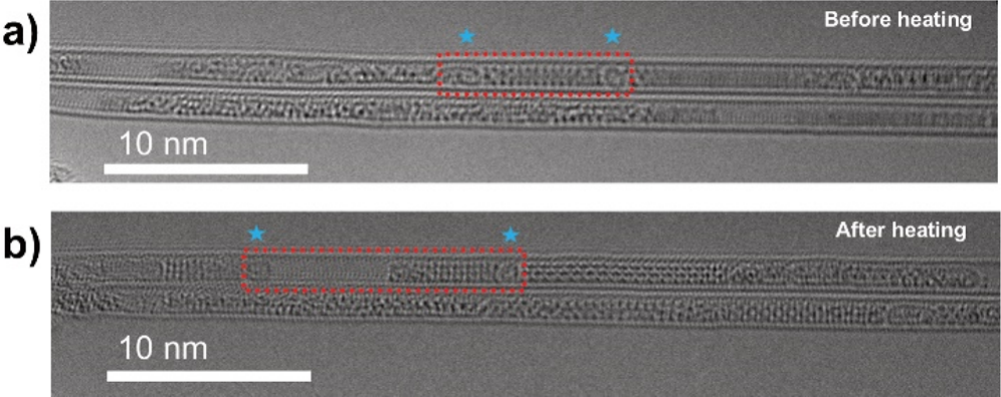
AC-TEM analysis of β-In_2_Se_3_ before and after heating to 400 °C. (a, b) AC-TEM images of a β-In_2_Se_3_ nanoribbon after heating to 23 and 400 °C, respectively. Red boxes represent the nanoribbon of interest, before and after heating. Blue stars are positioned above two fullerene-like molecules which “cap” the nanoribbon of interest.

At 400 °C a visible change in the structure of the encapsulated nanoribbons occurred, with the AC-TEM images of before and after heating being shown in Figure 6. Additionally, the mobility of the encapsulated nanoribbons was found to drastically decrease following the heating of the nanoribbon. In order to better understand the nature of this transformation, a small section of nanoribbon was chosen for further analysis, as outlined by red boxes in Figure 6. Serendipitously, two fullerene-like particles of amorphous carbon, ubiquitously present inside the SWCNTs as a byproduct of their synthesis and labeled with blue stars in Figure 6, were found to be “capping” a small nanoribbon of β-In_2_Se_3_, which could be used as reference points before and after heating.

**Figure 7:**
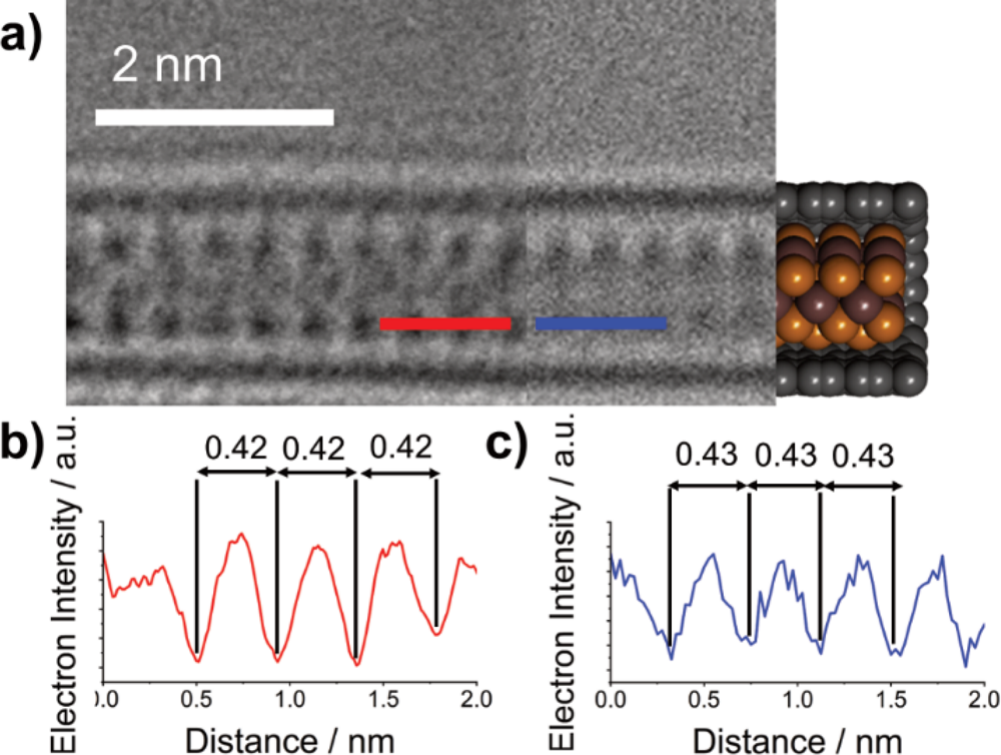
(a) Three-part composite image of a β-In_2_Se_3_ nanoribbon inside a SWCNT viewed along the (100) plane, rotated 10° in the axis of the SWCNT, consisting of an AC-TEM image (left), a simulated TEM image (center), and a molecular model (right) (b, c) Electron density profile maps in red, generated from the red line superimposed over the experimental AC-TEM image in (a), and in blue, generated from the blue line superimposed over the simulated TEM image in (b), with calculated interatomic distances highlighted in nm.

As shown in Figure 5, the nanoribbon observed at low temperature is known to be a monolayer extension of β-In_2_Se_3_. The orientation of this low-temperature phase nanoribbon was determined to be a monolayer of β-In_2_Se_3_ viewed along the (100) plane, rotated 10° in the axis of the SWCNT. Figure 7a–c shows the lower temperature nanoribbon seen in the experimental AC-TEM images compared to simulated TEM images, giving a very similar contrast pattern and nanoribbon diameter in projection. The process in which the crystal structure of bulk β-In_2_Se_3_ was truncated to create a nanoribbon is shown in Figure S8. The dimensions of the unit cell of bulk β-In_2_Se_3_ are reduced to encompass a monolayer of β-In_2_Se_3_, affording a “repeat unit” from which to build longer nanoribbons of monolayer β-In_2_Se_3_. It is important to note that the repeat unit cannot be classed as a unit cell in the traditional sense, as propagation in the *c* axis is impossible, due to the geometric constraints of the SWCNT.

**Figure 8:**
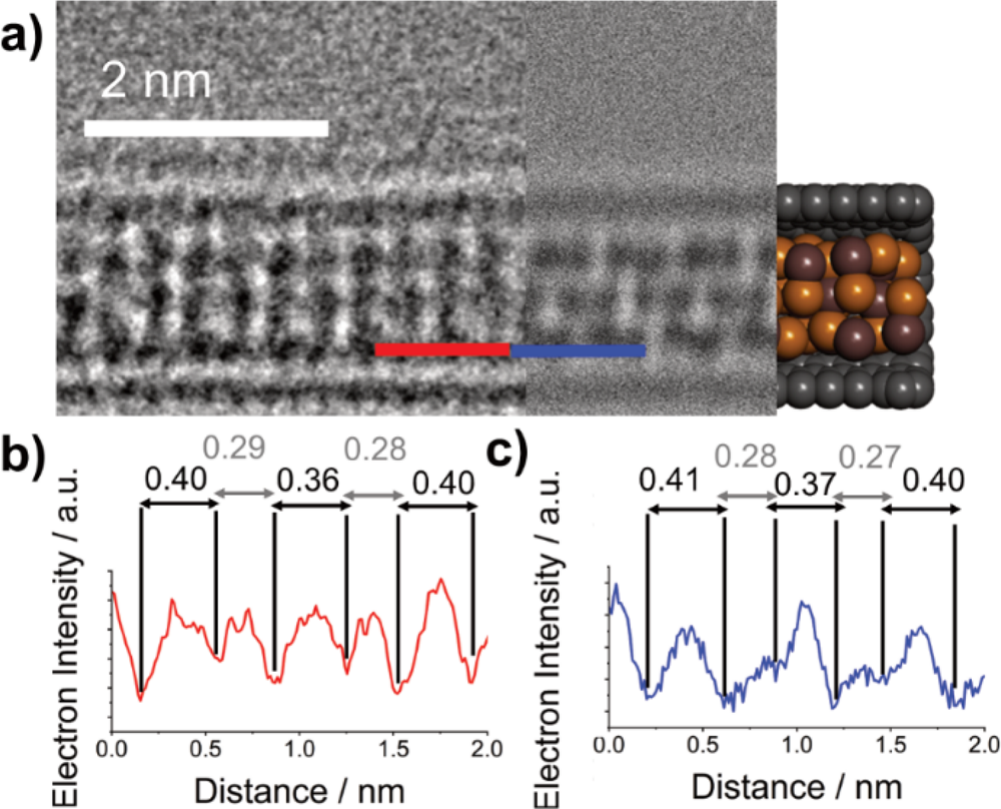
(a) Three-part composite image of a γ-In_2_Se_3_ nanowire inside a SWCNT viewed along the (120) plane, consisting of an AC-TEM image (left), a simulated TEM image (center), and a molecular model (right). (b, c) Electron density profile maps in red, generated from red line superimposed over the experimental AC-TEM image in (a), and in blue, generated from the blue line superimposed over the simulated TEM image in (b), with calculated interatomic distances highlighted in nm.

In order to eliminate the possibility that the changes in structure induced by heating were simply a rotation of the β-In_2_Se_3_ nanoribbon in the axis of the SWCNT, a rotational series simulating the rotation of β-In_2_Se_3_ viewed along the (100) plane was produced (Figure S9). No projections of the rotated (100) plane β-In_2_Se_3_ match the appearance of the nanoribbons seen at high temperatures (Figure 7b), indicating a thermally induced phase change has occurred. Bulk β-In_2_Se_3_ is known to undergo an irreversible phase transition to γ-In_2_Se_3_ at high temperatures. The exact temperature required for this transformation is contentious but has been reported to occur between 350 and 650 °C.^9,10^ We hypothesized that the same phase transition is seen here, transforming nanoconfined β-In_2_Se_3_ to γ-In_2_Se_3_. With this in mind, the crystal structure of bulk γ-In_2_Se_3_ was used as a starting point for creating a nanowire of the correct diameter and a similar appearance in projection to the higher temperature nanowire highlighted in Figure 6b. It was found that simulated TEM images of a nanowire constructed from γ-In_2_Se_3_ viewed along the (120) plane had a complementary appearance to the experimental AC-TEM images, as shown in Figure 8a–c. Once again, a “repeat unit” for a nanowire of γ-In_2_Se_3_ was constructed from the truncated crystal structure of bulk γ-In_2_Se_3_, as shown in Figure S10. It is important to note that because bulk γ-In_2_Se_3_ has a lattice in which atoms are covalently bonded in all three dimensions, the resultant encapsulated structures of γ-In_2_Se_3_ should be viewed as nanowires, as opposed to nanoribbons constructed from a single layer of β-In_2_Se_3_.

**Figure 9:**
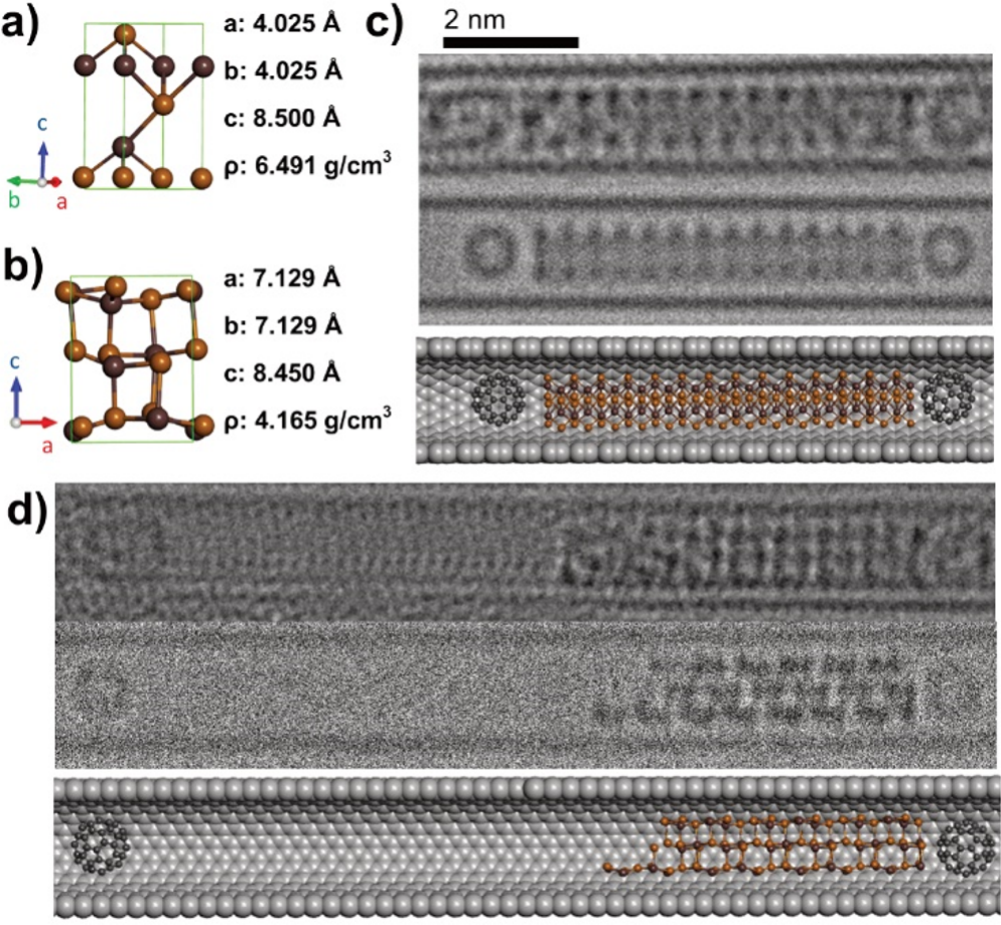
(a, b) The “repeat unit” used to create encapsulated structures of β-In_2_Se_3_ and γ-In_2_Se_3_, respectively. (c, d) Composite images comparing experimental AC-TEM images (top), simulated TEM images (middle), and molecular models (bottom) of the low-temperature phase β-In_2_Se_3_ and the high-temperature phase γ-In_2_Se_3_ respectively.

With the assumption that the two “capping” fullerene-like molecules prevented any additional Se or In atoms from neighboring nanowires from bonding to the existing nanowire during the heating experiment, the number of atoms in each nanoribbon/nanowire before and after heating could be assumed to be constant. Knowing this, the two repeat units used for simulating the β-In_2_Se_3_ nanoribbon and the γ-In_2_Se_3_ nanowire, shown in Figure 9a,b, were extended into 2D sheets. From these 2D sheets a nanoribbon and a nanowire, both containing 140 atoms (84 Se and 56 In), were constructed, with the processes being shown in Figures S11 and S12. These structures were created to have the same appearance in projection as those shown above (Figures 7 and 8), while possessing various custom-made defect sites to match experimental AC-HRTEM images (Figure 6). In both the low-temperature β-In_2_Se_3_ nanoribbon and the high-temperature γ-In_2_Se_3_ nanowire the dimensions of the simulated structures closely match the experimental TEM images. The change in nanowire volume following heating is further evidence that a phase change has occurred.

Overall, we have observed a thermally induced phase transition in direct space, at the atomic level, changing a β-In_2_Se_3_ nanoribbon to a γ-In_2_Se_3_ nanowire. It is important to emphasize the value of local-probe analysis for determining the ability of these sub-nm-wide indium selenides to change phase. DSC measurements were attempted (Figure S13) on β-In_2_Se_3_@SWCNT powder but did not yield a meaningful result. Additionally, variable-temperature Raman analysis (Figure S14) was attempted, monitoring the RBM position of β-In_2_Se_3_@SWCNT and empty SWCNTs from room temperature to 400 °C, but showed no significant evidence of a phase change occurring. This is likely due to encapsulated In_2_Se_3_ nanoribbons/nanowires having a variety of lengths and propagation regimes and therefore a variety of phase-change temperatures and thermal expansion coefficients.^37,58−60^ This indicates that analytical methods averaging over an ensemble of nanoribbons of different lengths/propagation regimes cannot be informative for understanding the phase transitions in these materials. However, evidence for this phase transition being reversible can be inferred from the synthesis of β-In_2_Se_3_@SWCNT. If the transition was irreversible, then only γ-In_2_Se_3_ would be seen inside SWCNTs, as it would be formed during the high-temperature annealing stage (550 °C, Ar, 1 h) of this synthesis. Because of this, it is expected that γ-In_2_Se_3_ is only metastable with respect to the β-In_2_Se_3_ phase (proposed reaction coordinate diagrams are shown in Figure S15).

## Conclusions

Indium selenides are an exciting family of semiconductors with complex polymorphism and a variety of desirable properties, including ferroelectricity, phase-dependent optical properties, and high electron mobilities, when confined to two dimensions. Often heralded as a potential component in future nanoelectronic devices, a greater understanding of how to synthesize nanosized In_*x*_Se_*y*_ is clearly needed.

In this study we designed two approaches for the growth of monolayer, sub-nm-wide In_*x*_Se_*y*_ nanoribbons by using SWCNTs as a template. The first of these, performed by melting solid γ-InSe into SWCNTs, yielded monolayer InSe nanoribbons with a width of 0.67 nm. The second, a stepwise growth from Se and InCl precursors, yielded monolayer β-In_2_Se_3_ nanoribbons with a width of 0.85 nm. Carbon nano test tubes offered an opportunity to study the complex polymorphism of β-In_2_Se_3_ nanoribbons via AC-HRTEM, identifying a thermally induced phase change to a γ-In_2_Se_3_ nanowire following heating to 400 °C. Overall, this study provides a robust synthetic method for the production of two different phases of sub-nm-wide In_*x*_Se_*y*_ nanoribbons, while also confirming that the desirable phase change behavior of bulk In_*x*_Se_*y*_ is retained when the material is reduced to this diameter. Our work on controlling the phase of sub-nm In_*x*_Se_*y*_ nanoribbons offers an opportunity to produce bespoke and versatile nanoelectronic devices in the future.

## Experimental Section

### Materials

SWCNTs (P2-SWCNTs, arc-discharge, Carbon Solutions, USA) were refluxed in concentrated hydrochloric acid for 1 h to remove residual metal catalyst from their synthesis. This was followed by annealing at 600 °C for 17 min to open and remove end caps and most of the amorphous carbon from their internal cavities and external surfaces, resulting in a 50% mass loss. Selenium (Sigma-Aldrich) and indium monochloride (Sigma-Aldrich) were used as received. γ-InSe was synthesized in-house using the Bridgman method from a polycrystalline melt of In and Se, as described by Mudd et al.^14^

### Growing InSe@SWCNTs from Bulk γ-InSe

Opened P2 SWCNT (5 mg) and γ-InSe (5 mg) were sealed under vacuum (10^–5^ mbar) in a quartz ampule and heated to 800 °C for 70 h. The product was then cooled under vacuum and removed from the ampule for analysis, giving InSe@SWCNTs (7.5 mg).

### Growing β-In_2_Se_3_@SWCNTs from Se and InCl

Opened P2 SWCNTs (30 mg) and Se (30 mg) powder were sealed under vacuum (10^–5^ mbar) in a Pyrex ampule and heated to 550 °C for 70 h. The resultant material was then washed with CS_2_, giving Se@SWCNTs (35 mg). Se@SWCNTs (30 mg) and InCl (30 mg) were sealed under vacuum (10^–5^ mbar) in a Pyrex ampule and heated to 350 °C for 70 h. The resultant material was then cleaned by heating one end of the ampule to 380 °C to remove excess InCl, followed by sonication in concentrated HCl under a flow of Ar gas (15 mL/min) for 1 h to remove externally bound In_2_O_3_, giving “Se+InCl”@SWCNTs (10 mg). “Se+InCl”@SWCNTs (4 mg) were sealed in a Pyrex ampule under argon (15 Hg/mm) and heated to 550 °C for 1 h, giving β-In_2_Se_3_@SWCNTs (4 mg).

### Electron Microscopy

Samples were suspended in propan-2-ol and drop-cast onto lacey-carbon-coated copper TEM grids. TEM imaging was conducted using a JEOL 2100F FEG-TEM microscope operated 200 kV. AC-TEM was performed using a *C*_s_-corrected SALVE TEM microscope operated at 60 kV at the University of Ulm and a JEOL ARM200CF instrument optimized for atomic resolution spectroscopy operated at 80 kV at the electron Physical Science Imaging Centre (ePSIC). For in situ TEM heating experiments samples were suspended in propan-2-ol and drop-cast onto a DENS Solutions heating chip. The electron beam was blanked during heating to avoid any electron-beam-induced phase transitions occurring.

### Energy Dispersive X-ray Spectroscopy

Local EDX spectra were acquired for samples mounted on lacey-carbon-coated copper TEM grids using an Oxford Instruments INCA X-ray microanalysis system.

### TEM Simulation

TEM image simulations were carried out using QSTEM, a multislice program which uses the Dirac–Fock scattering potential of Rez et al.^41,61^ A fixed number of 20 slices per nanotube was chosen, and images were calculated with a sampling set to match experimental conditions. The defocus and aberration parameters were set according to the values used in experimental imaging. The effect of limiting electron dose to the images was conducted using a custom-made Monte Carlo program that applies noise by utilizing the Poisson statistics of electrons.

### X-ray Photoelectron Spectroscopy

X-ray photoelectron spectroscopy (XPS) measurements were carried out using a Kratos AXIS ULTRA instrument with a monochromatic Al Kα X-ray source (1486.6 eV) at 12 kV anode potential (120 W) and 10 mA emission current.

### Raman Spectroscopy

Micro Raman spectroscopy was performed using a HORIBA LabRAM HR Raman microscope. Spectra were acquired using a 660 nm laser (at ∼0.1 mW (1%) power), a 100× objective and a 200 μm confocal pinhole. To simultaneously scan a range of Raman shifts, a 600 lines mm^–1^ rotatable diffraction grating along a path length of 800 mm was employed. Spectra were detected using a Synapse CCD detector (1024 pixels) thermoelectrically cooled to −60 °C. Before spectra collection, the instrument was calibrated using the zero-order line and a standard Si(100) reference band at 520.7 cm^–1^. The spectral resolution is better than 1.2 cm^–1^ in this configuration.

Variable-temperature measurements were performed within a Linkam THMS600 stage. A typical experiment was as follows: the spectrum was collected at 25 °C, the temperature was then increased at a rate of 20 °C/min to 50 °C and held for 2.5 min for equilibration, the sample surface was found by manual refocusing (*xy*) and depth profiling (*z*), and then a further spectrum was collected. This procedure was repeated in 25 °C steps up to 500 °C.

### Thermogravimetric Analysis

A TA Q500 Thermogravimetric Analyzer was used for the thermogravimetric analysis. All samples were analyzed using a platinum pan and in the presence of air. Experimental parameters were as follows: 10 min isothermal hold at room temperature, ramp from room temperature to 1000 °C at 10 °C/min, followed by a final 10 min isothermal hold at 1000 °C.

### Powder X-ray Diffraction

A PANalytical X’Pert Pro diffractometer was used for the powder X-ray measurements. This was achieved using a Cu Kα radiation source (λ = 1.5432 Å, 40 kV 40 mA) in a Bragg–Brentano geometry on a Si zero-background holder. The parameters for a typical experiment were the following: 0.0525° step size, 0.00220°/s scan speed, 5° start angle, 80° stop angle, and 6080 s time/step.

### Differential Scanning Calorimetry

Differential scanning calorimetry (DSC) experiments were conducted on a TA Instruments Discovery DSC2500 instrument equipped with an RCS-90 chiller using 3 mg of sample. Dry nitrogen gas was used for all experiments at a flow rate of 50 mL min^–1^. Samples were prepared in sealed aluminum Tzero hermetic pans.

## Supporting Information Available

Further data including XPS, PXRD, TEM, DSC, and variable-temperature Raman spectroscopy, molecular models describing the nanoribbon and nanowire building process, and estimated reaction coordinate diagrams (PDF)
