# Supplementary material for: Subnanometer-Wide Indium Selenide Nanoribbons
Source: ACS Nano. 2023 Mar 14;17(6):6062–72. doi: 10.1021/acsnano.3c00670 (PMC10061931; doi:10.1021/acsnano.3c00670)
Supplement: Supplementary file 1 — nn3c00670_si_001.pdf [file nn3c00670_si_001.pdf]

Sub-nanometer Wide Indium Selenide Nanoribbons

Supporting Information

William J. Cull,[a] Stephen T. Skowron,[a] Ruth Hayter,[a] Craig T. Stoppiello,[b,] Graham A. Rance,[b] Johannes Biskupek,[c] Zakhar R. Kudrynskyi,[d,e] Zakhar D. Kovalyuk,[f] Christopher S. Allen,[g] Thomas J. A. Slater,[g] Ute Kaiser,[c] Amalia Patanè[d] and Andrei N. Khlobystov*[a,b]

a: School of Chemistry, University of Nottingham, Nottingham, NG7 2RD, United Kingdom.

b: Nanoscale and Microscale Research Centre, University of Nottingham, Nottingham, NG7 2QL, United Kingdom.

c: Central Facility of Electron Microscopy, Electron Microscopy Group of Materials Science, University of Ulm, 89081, Ulm, Germany.

d: School of Physics, University of Nottingham, Nottingham, NG7 2RD, United Kingdom.

e: Faculty of Engineering, University of Nottingham, Nottingham, NG7 2RD, United Kingdom.

f: Institute for Problems of Materials Science, National Academy of Sciences of Ukraine, Chernivtsi Branch, 58001 Chernivtsi, Ukraine.

g: Electron Physical Sciences Imaging Centre, Diamond Light Source ltd, Didcot, OX11 0DE, United Kingdom.

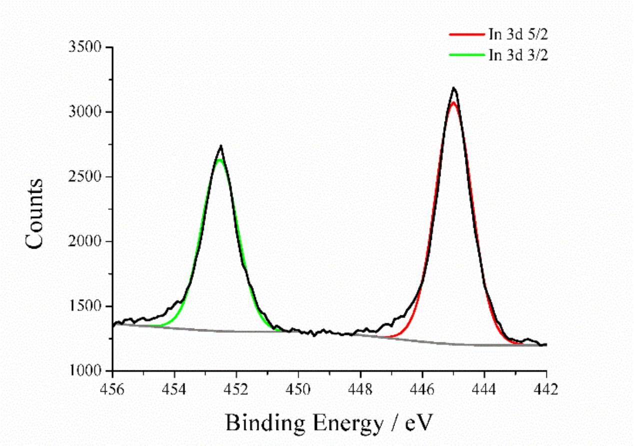

Figure S1. XPS analysis showing In 3d photoelectron lines for InSe@SWCNT (melt growth method)

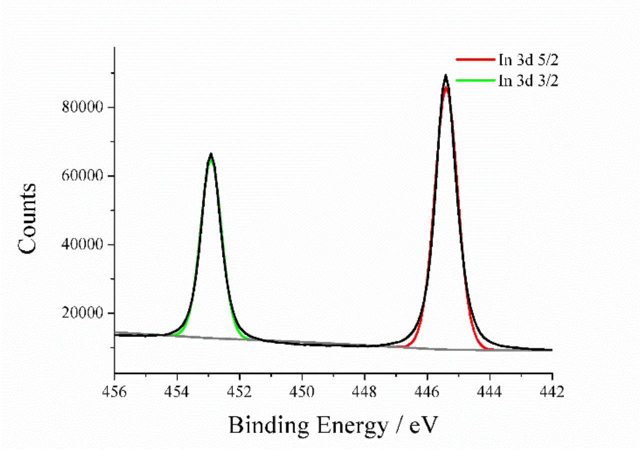

Figure S2. XPS analysis showing In 3d photoelectron lines for bulk γ-InSe

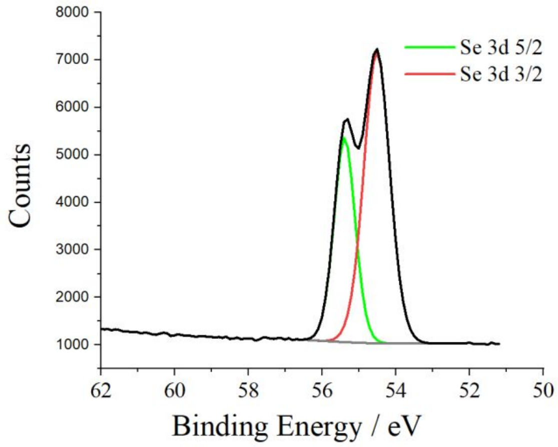

Figure S3. XPS analysis showing Se 3d photoelectron lines for bulk γ-InSe

a) b)

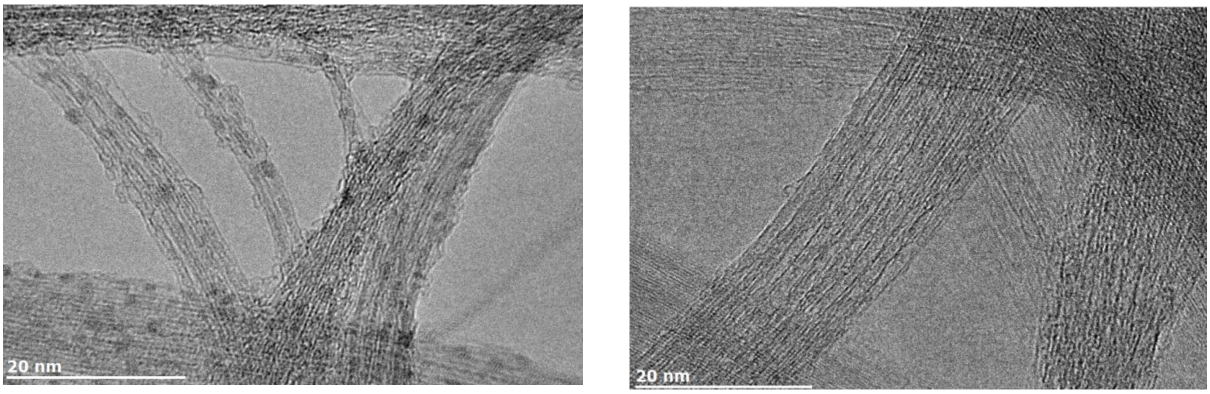

Figure S4. TEM analysis of β-In_2Se_3@SWCNT (sublimation filling method) before (a) and after (b) cleaning steps. The externally bound InCl particles are removed from the SWCNTs following the cleaning procedure

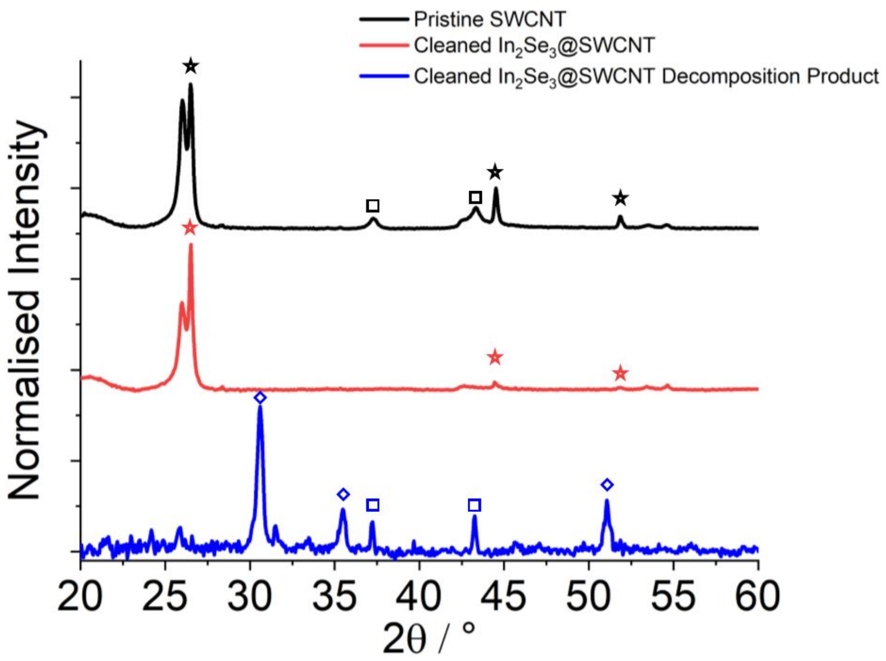

Figure S5. PXRD Analysis of cleaning of β-In_2Se_3@SWCNT. Stars are known SWCNT diffraction peaks,[1] Squares are known NiO diffraction peaks[2] and diamonds are known In_2O_3 diffraction peaks.[3]

CNT: Stars, (002) at 26.6, (100) at 44.5, (004) at 51.8

NiO: Squares, (111) at 37.3, (020) at 43.3,
10.2183/pjab.55.43

In_2O_3: Diamonds: (222) at 30.6, (040) at 35.5, (044) at 51.1
10.1107/S0365110X66001749

$$CNT + nO_2 \rightarrow \mathbf{CO_2}_{\mathbf{(g)}}$$

$$In_2Se_3 + 4\,½\,O_2 \rightarrow \mathbf{In_2O_3}_{\mathbf{(s)}} + 3\mathbf{SeO_2}_{(g)}$$

$$Ni + ½\,O_2 \rightarrow \mathbf{NiO}_{\mathbf{(s)}}$$

Equation S1. Balanced equation for the complete combustion of β-In_2Se_3@SWCNT In air

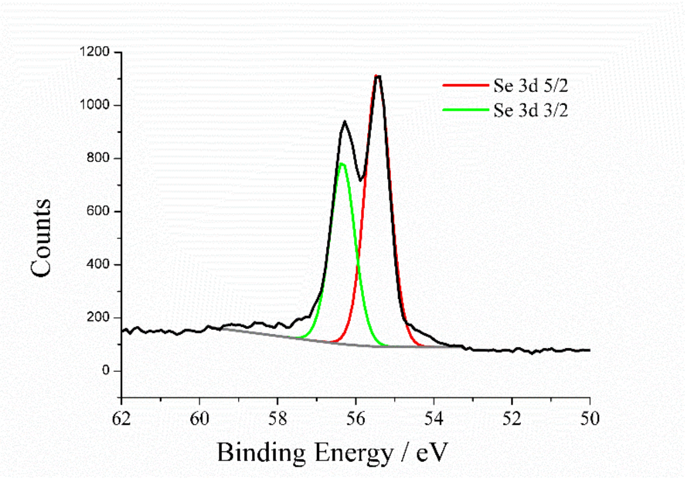

Figure S6. XPS analysis showing Se 3d photoelectron lines for Se@SWCNT

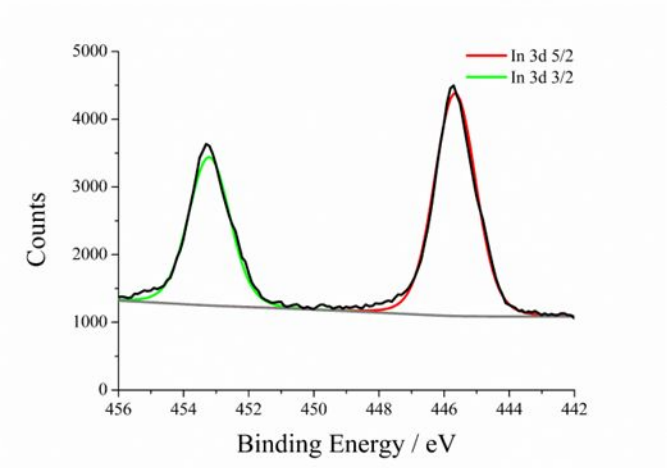

Figure S7. XPS analysis showing In 3d photoelectron lines for β-In_2Se_3@SWCNT

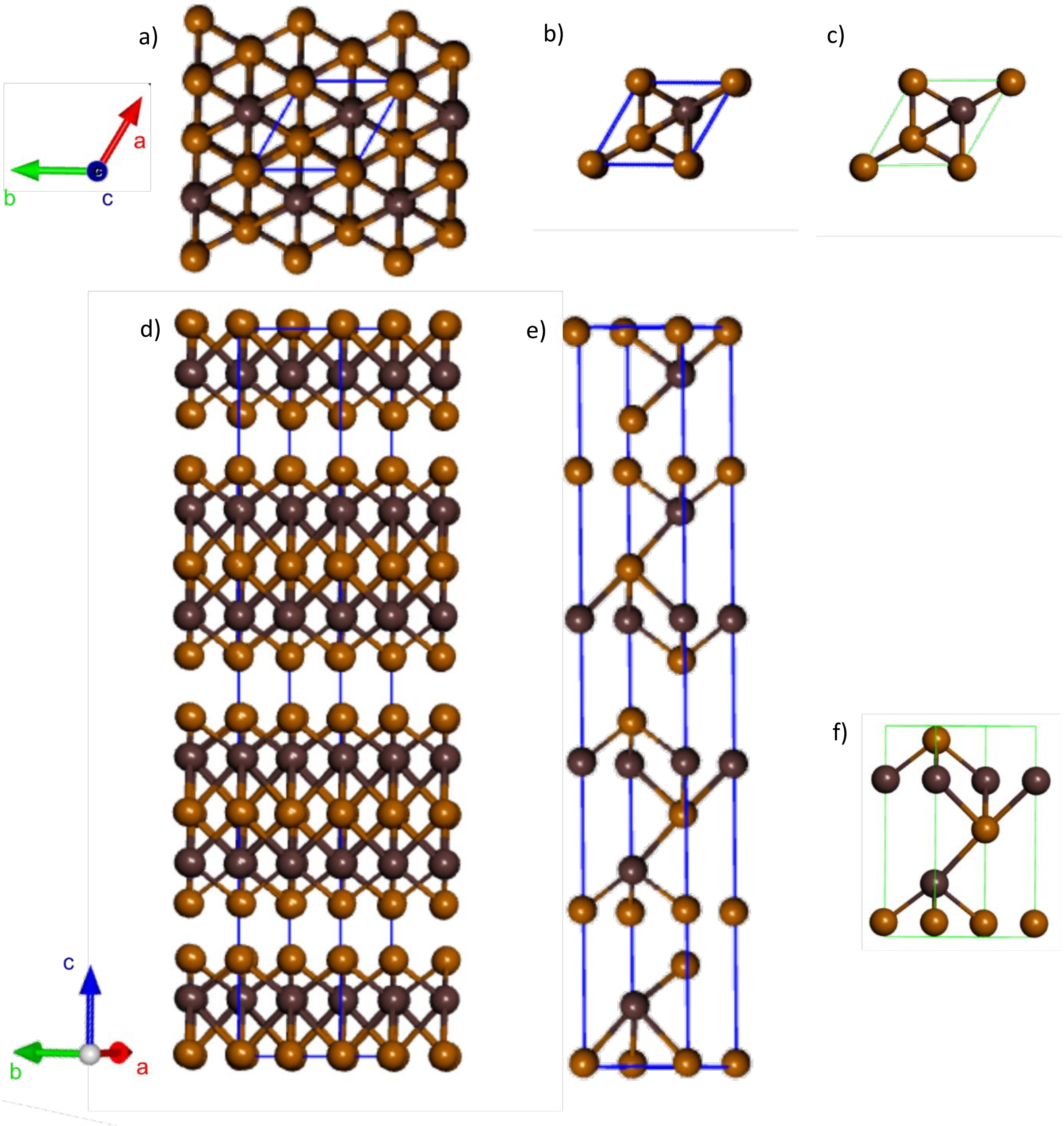

Figure S8. Truncation of Bulk β-In_2Se_3 to it's unit cell, followed by truncation to a β-In_2Se_3 nanoribbon repeat unit. Unit cells highlighted in blue, 'repeat unit' cells highlighted in green. a) (001) plane bulk β-In_2Se_3, b) (001) plane β-In_2Se_3 unit cell, c) (001) plane β-In_2Se_3 nanoribbon 'repeat unit', d) (100) plane bulk β-In_2Se_3, e) (100) plane β-In_2Se_3 unit cell f) (100) plane β-In_2Se_3 nanoribbon 'repeat unit'.

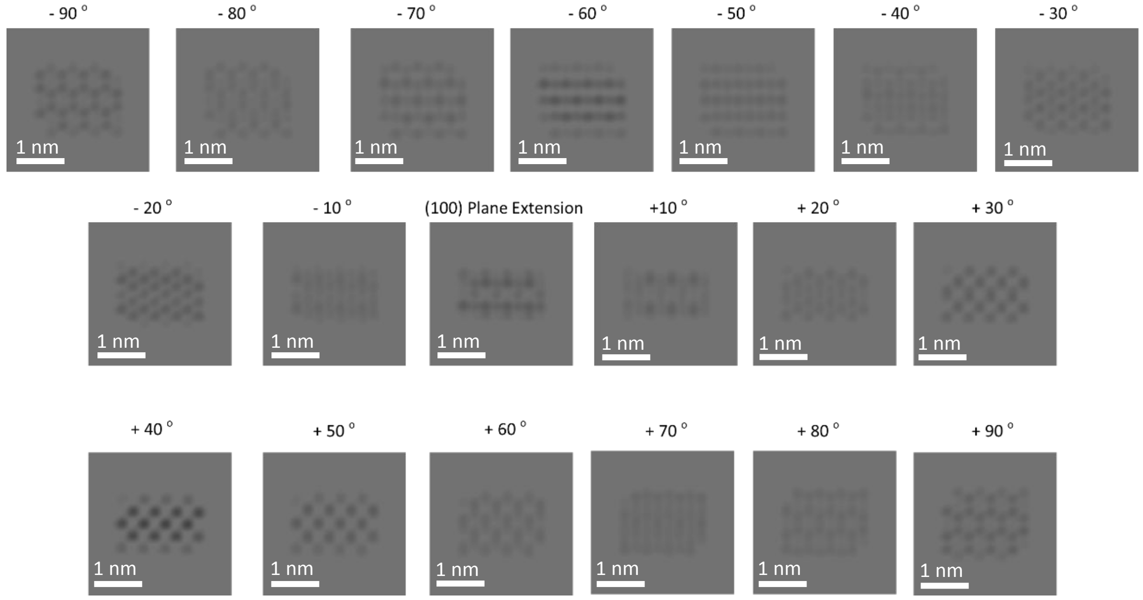

Figure S9. Rotational tableau of (100) plane β-In_2Se_3 simulated using QSTEM. The +10 ° orientation is seen in fig. 7a and the +40° rotation is seen in fig. 5f.

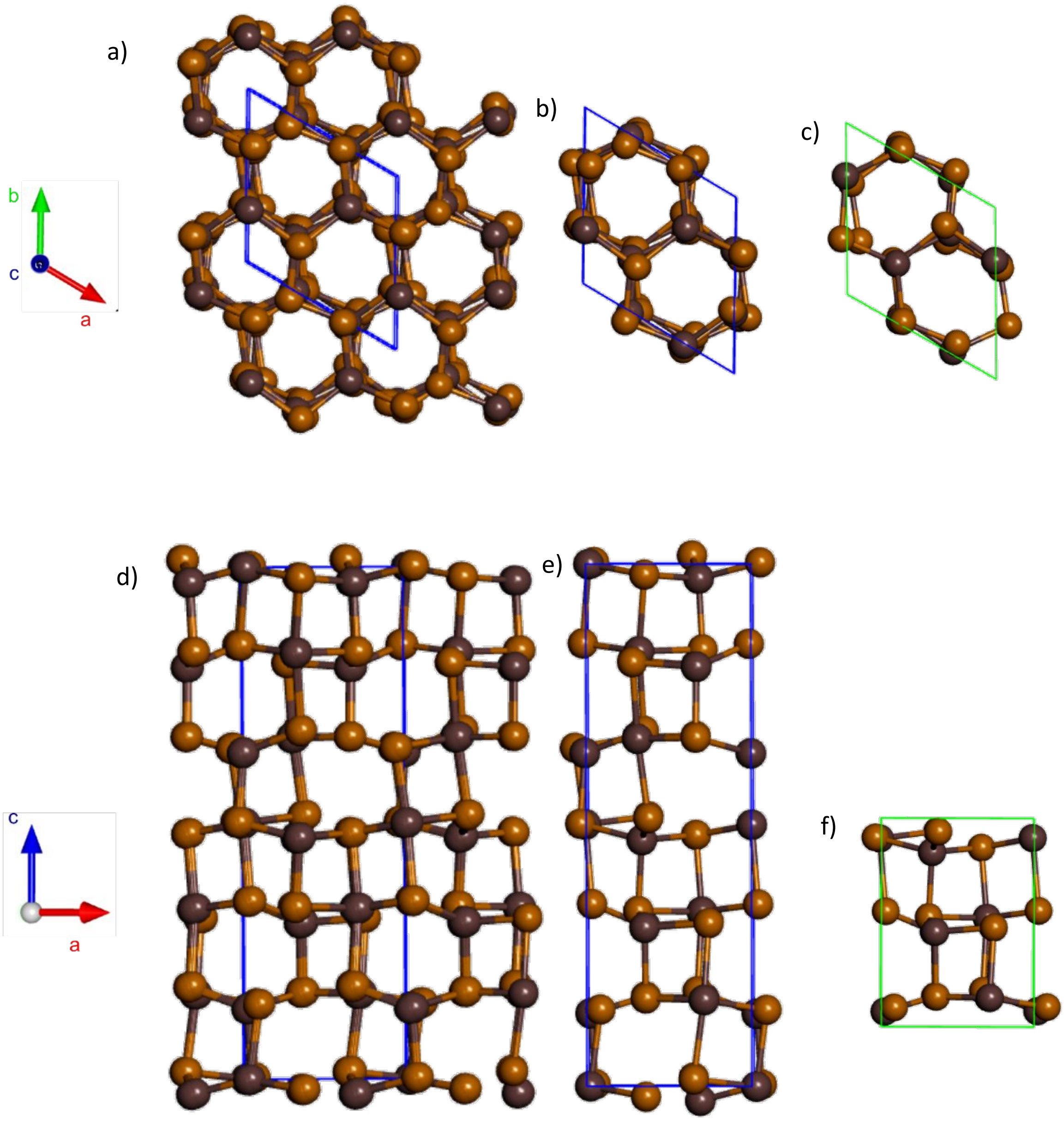

Figure S10. Truncation of Bulk γ -In_2Se_3 to it's unit cell, followed by truncation to a γ-In_2Se_3 nanowire repeat unit. Unit cells highlighted in blue, 'repeat unit' cells highlighted in green. a) (001) plane bulk γ-In_2Se_3, b) (001) plane γ-In_2Se_3unit cell, c) (001) plane γ-In_2Se_3 nanowire 'repeat unit', d) $(\bar{1}20)$ plane bulk γ-In_2Se_3, e) $(\bar{1}20)$ plane γ-In_2Se_3unit cell f) $(\bar{1}20)$ plane γ-In_2Se_3 nanowire 'repeat unit'.

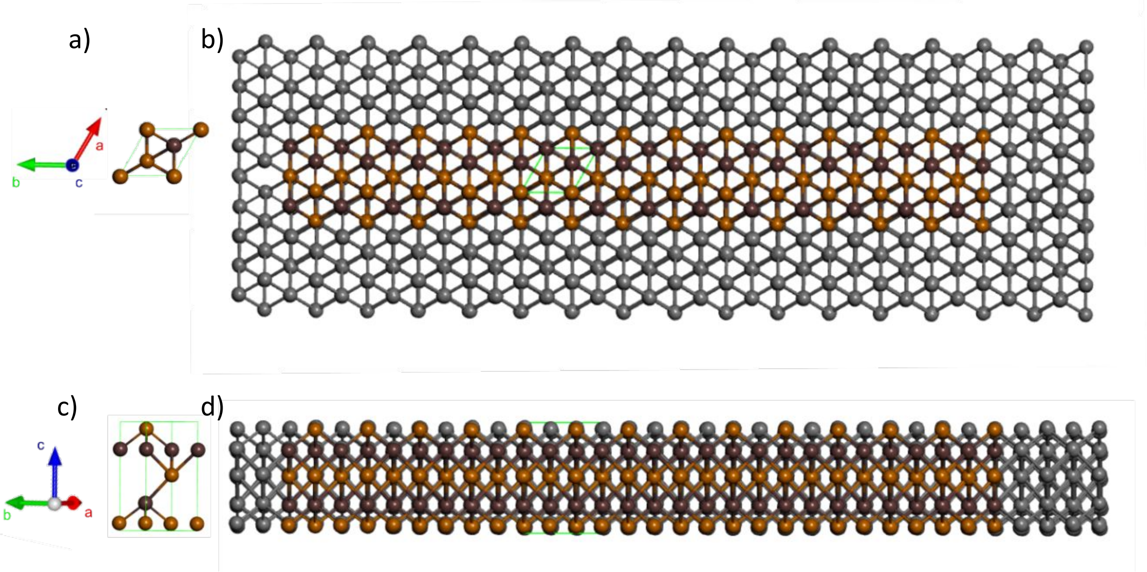

Figure S11. Creation of a β-In_2Se_3 nanoribbon (CPK coloured) from a 2D sheet of β-In_2Se_3 (Grey). 'Repeat unit' cell shown in green. a) (001) plane β-In_2Se_3 nanoribbon 'repeat unit', b) (001) plane β-In_2Se_3 nanoribbon, c) (100) plane β-In_2Se_3 nanoribbon 'repeat unit', d)) (100) plane β-In_2Se_3 nanoribbon.

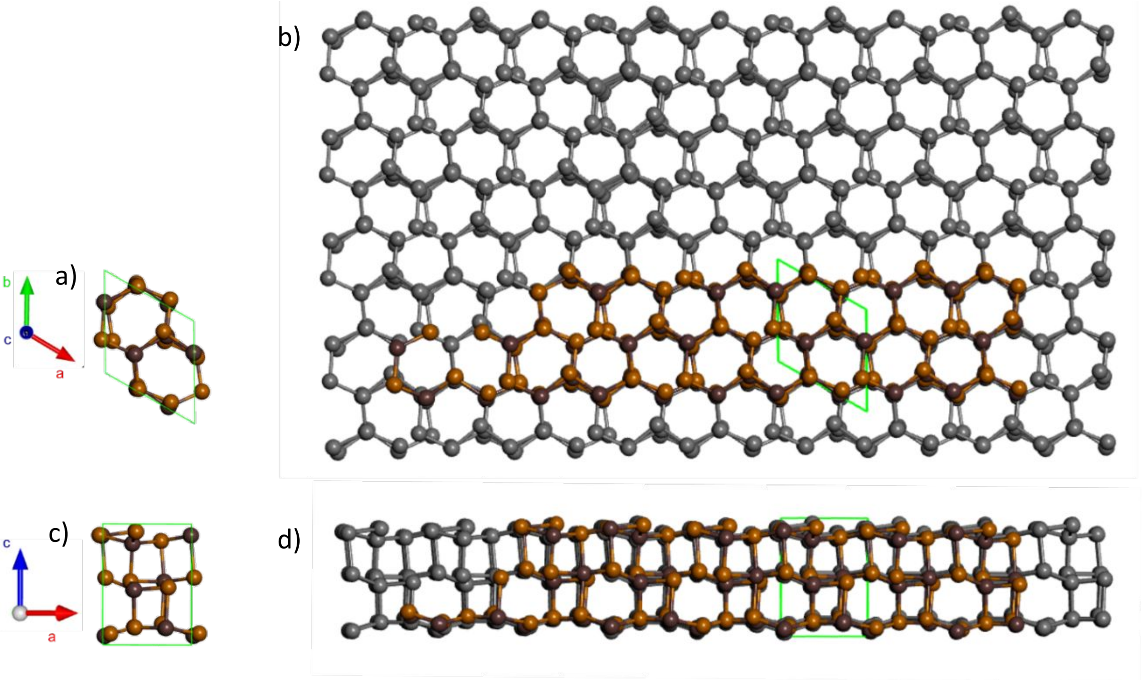

Figure S12. Creation of a γ-In_2Se_3 nanowire (CPK coloured) from a 2D sheet of γ-In_2Se_3 (Grey). 'Repeat unit' cell shown in green. a) (001) plane γ-In_2Se_3 nanowire 'repeat unit', b) (001) plane γ-In_2Se_3 nanowire, c) $(\bar{1}20)$ plane γ-In_2Se_3 nanowire 'repeat unit', d) $(\bar{1}20)$ plane γ-In_2Se_3 nanowire.

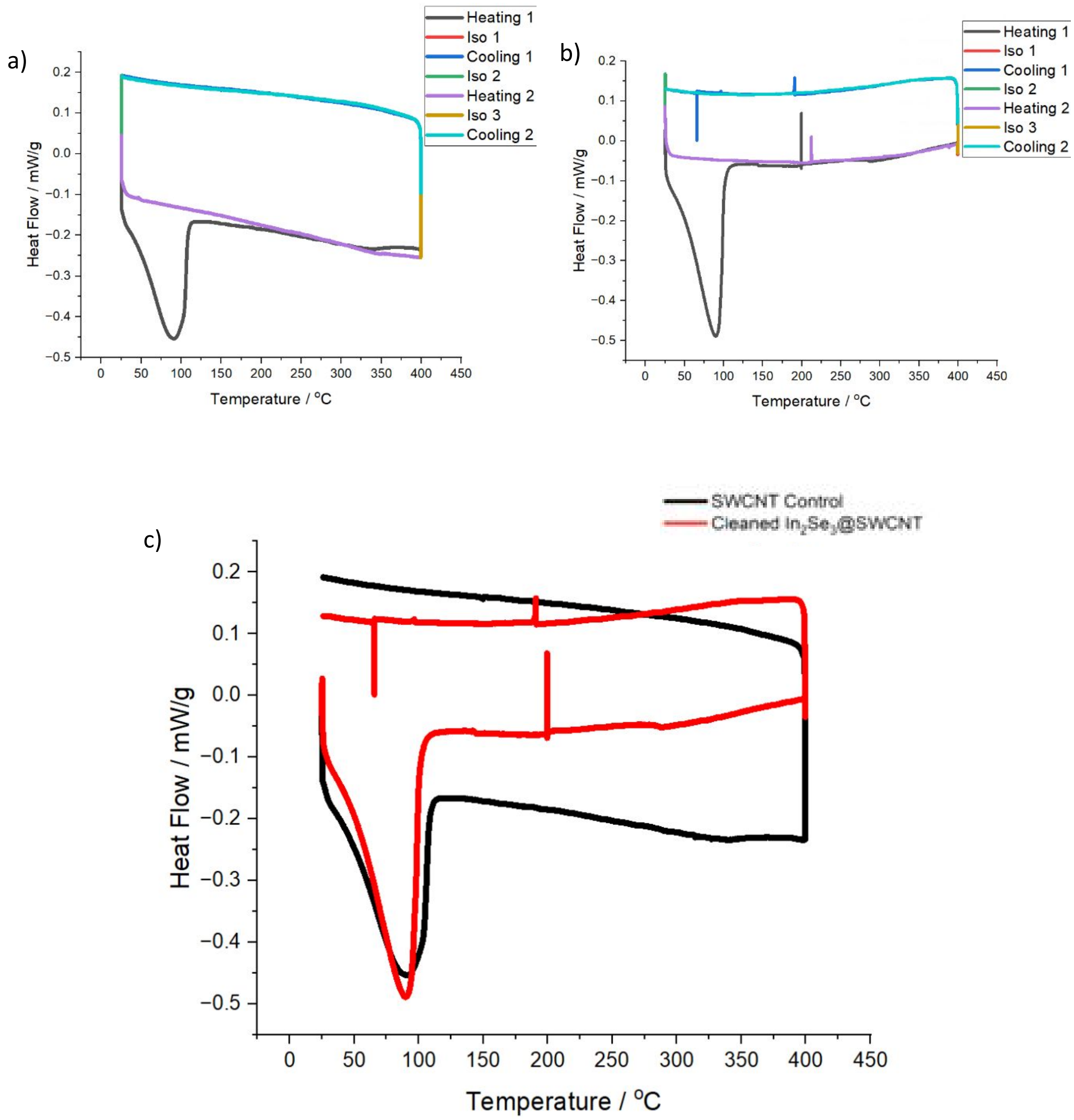

Figure S13. DSC analysis of β-In_2Se_3@SWCNT and SWCNT Control. a) DSC thermogram for Control SWCNTs, b) DSC Thermogram for cleaned In_2Se_3@SWCNT, c) The first heating an cooling cycle in the thermograms of a) and b) overlayed on one another.

In order to further study the reversibility of this phase change DSC analysis of β-In_2Se_3@SWCNT and empty control SWCNTs was performed, with results shown in fig. S13. Each sample was heated from room temperature to 400 °C, then cooled to room temperature, twice, with a 10 minute isothermal in between each heating/cooling period. Unfortunately, DSC analysis was unable to provide sharp peaks corresponding to phase transitions from the encapsulated material. However, it can be noted that the filled SWCNTs experienced a shallow, endothermic peak when compared to control SWCNTs upon heating. This process also appeared to be repeatable. The only peak of note in both thermograms is the irreversible water loss seen at around 100 °C. It is possible that due to encapsulated In_2Se_3 NWs having a variety of lengths what would be a sharp phase transition in bulk In_2Se_3 is spread over a range of temperatures, due to nanoconfinement effects.

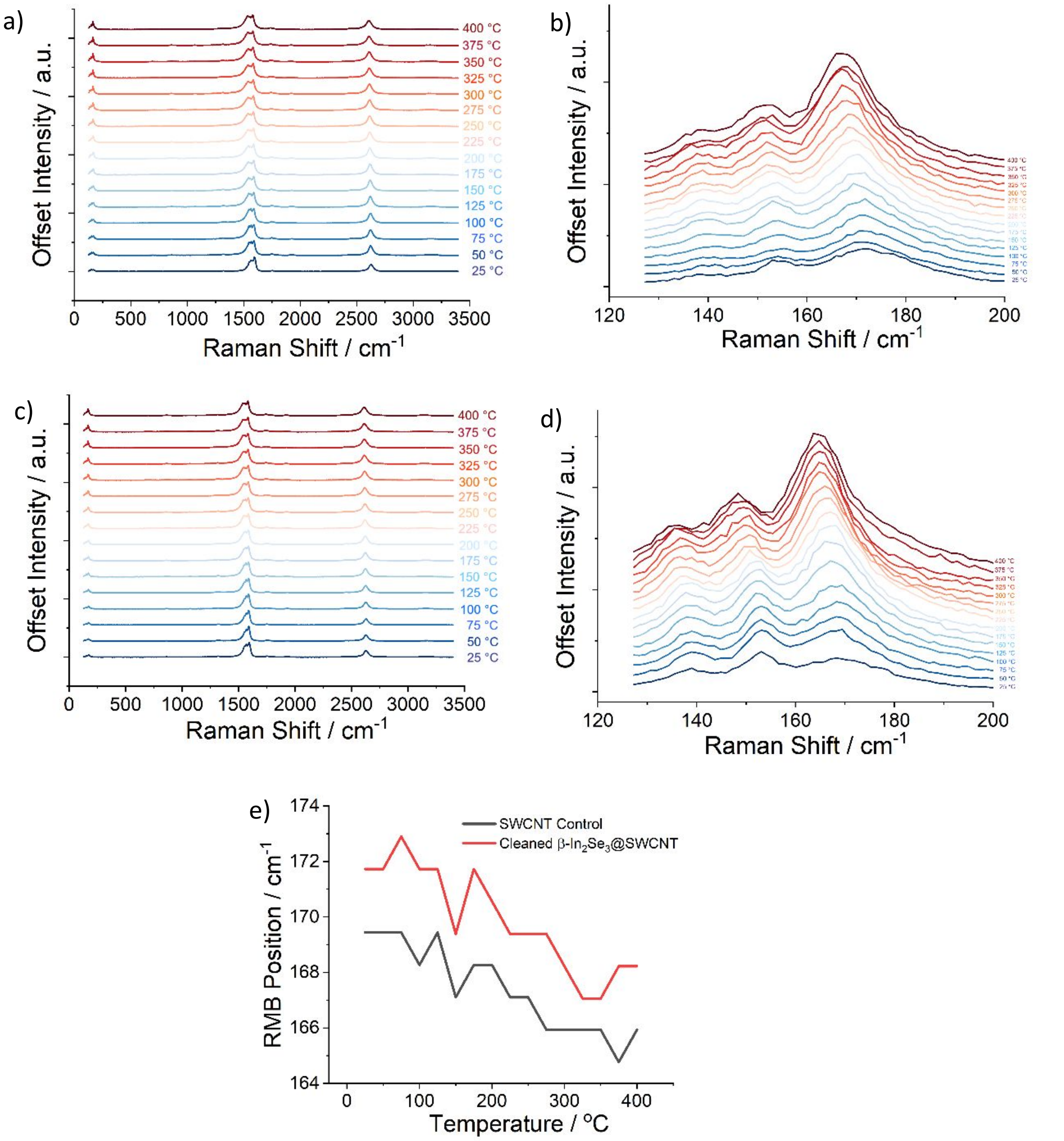

Figure S14. Variable temperature Raman analysis of filled and unfilled SWCNTs above and below the phase change temperature of nanoconfined β-In_2Se_3 to γ-In_2Se_3. a) and b) the full spectral window and RBM region, respectively, for β-In_2Se_3@SWCNT, c) and d) the full spectral window and RBM region, respectively, for control SWCNTs, e) the change in RBM position as a function of temperature for control SWCNTs (black) and β-In_2Se_3@SWCNT (red). All spectra have been normalised to the intensity of the G band of the SWCNT for display purposes.

There is a small difference in the relative position of the maximum RBM as a function of temperature but it is not significant and the trends are near co-linear with each other, providing no evidence for phase change. Similar to the DSC results, encapsulated inside the SWCNTs are nanoribbons of

various propagation directions, diameters and orientations. This results in a non-uniform change in the diameters of the SWCNT following the phase change from β-In_2Se_3 to γ-In_2Se_3, and therefore an insignificant overall change in the RBM when compared to control SWCNTs.

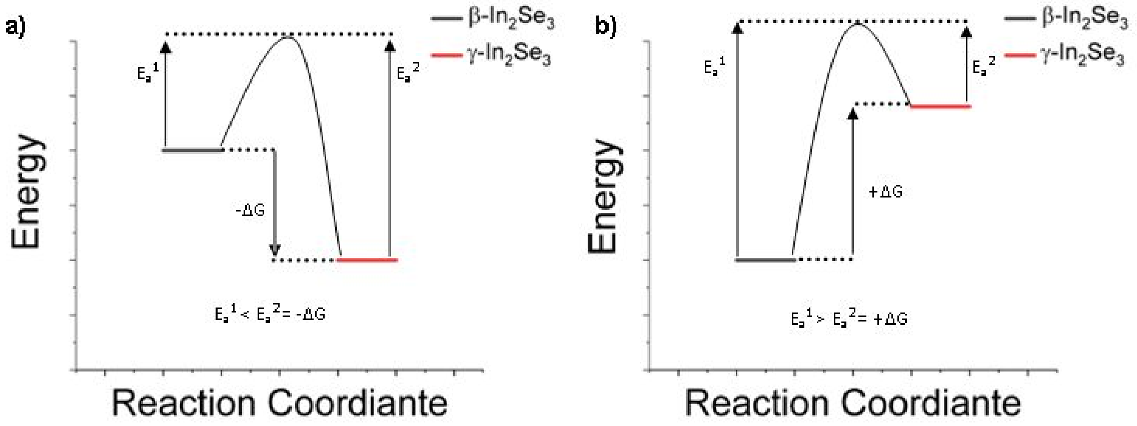

Figure S15. Proposed reaction coordinate diagrams for the thermally induced phase change of β-In_2Se_3 to γ-In_2Se_3. In a) β-In_2Se_3 is metastable with respect to γ-In_2Se_3 and in b) γ-In_2Se_3 is metastable with respect to β-In_2Se_3. E_a^1 is the activation energy required to thermally convert β-In_2Se_3 to γ-In_2Se_3, E_a^2 is the activation energy required to thermally convert γ -In_2Se_3 to β-In_2Se_3 and ΔG is the Gibbs free energy change accompanying the phase transition.

It is likely that the reaction coordinate diagram in fig. S15b better models the thermally induced phase change between β-In_2Se_3@SWCNT and γ-In_2Se_3@SWCNT, as if β-In_2Se_3 was only metastable with respect to γ-In_2Se_3 every single encapsulated nanowire would be irreversibly converted to γ-In_2Se_3 during the final high temperature annealing step (550 °C, 1 hr, Ar) of the synthesis.

References

1. Ngoma, M. N.; Mathaba, M.; Moothi, K. Effect of carbon nanotubes loading and pressure on the performance of a polyethersulfone (PES)/carbon nanotubes (CNT) membrane, *Sci. Rep.* **2021**, *11*, 1-12.
2. Sasaki, S.; Fujino, K.; Takéuchi, Y. X-Ray Determination of Electron-Density Distributions in Oxides, MgO, MnO, CoO, and NiO, and Atomic Scattering Factors of their Constituent Atoms, *Proc. Japan Acad. Ser. B* **1979**, *55*, 43-48.
3. Marezio, M.; Refinement of the Crystal Structure of In_2O_3 at two Wavelengths, *Acta Crystallogr.* **1966**, *20*, 723-728.
